# Improvement of egg internal quality of local ducks through star gooseberry leaf meal inclusion in ration 

**DOI:** 10.5455/javar.2023.j695

**Published:** 2023-09-24

**Authors:** Anggraeni Anggraeni, Deden Sudrajat, Ristika Handarini, Burhanudin Malik

**Affiliations:** Department of Animal Science, Faculty of Agriculture, Djuanda University, Bogor, Indonesia

**Keywords:** Sauropus androgynus, cholesterol, antioxidation, egg quality

## Abstract

**Objectives:**

The use of dried star gooseberry leaf extract (DSGLE) in rations and its effects on the egg internal quality of local ducks were examined.

**Materials and Methods:**

A total of 100 9-month-old local ducks weighing 1,406.25 ± 211.32 gm were randomly allocated into 5 treatments and 5 replicates in a completely randomized design. The birds were reared in 25 battery cages (4 birds each) and fed rations containing 0, 0.5%, 1.0%, 1.5%, and 2.0% DSGLE.

**Results:**

Eggs of ducks fed DSGLE had significantly lower saturated fatty acid (SFA) (30.66%–32.53%) than those of control ducks (36.23%). Egg polyunsaturated fatty acids (PUFA) increased from 2.29% (control) to 2.61% (2.0% DSGLE) and 2.76% (1.5% DSGLE), while egg monounsaturated fatty acid (MUFA) contents were not significantly different. The fatty acids of the whole edible part of eggs (albumen + yolk) were composed mainly of MUFA (40.19%–44.34%), followed by SFA (30.66%–36.22%), and PUFA (2.29%–2.76%). Malondialdehyde contents were reduced from 6.07 to 4.92 µg/gm (19%) in egg yolk and from 1.50 to 0.62 µg/gm (59%) in egg white, and thiobarbituric acid reactive substances values of the eggs were reduced from 0.93 to 1.65 (77%). The egg cholesterol level of ducks fed 2% DSGLE (21.94 mg/gm) was about 28.5% lower than that of eggs from control ducks (30.67 mg/gm) of eggs of control ducks. The egg white and egg yolk protein contents (12.31% and 16.35%) of treated ducks were lower than those in the control group (13.58% and 17.47%).

**Conclusion:**

The inclusion of SGLE in rations could be used to produce healthy duck eggs with no oxidative damage.

## Introduction

In recent years, with higher concern for a healthy life, consumers have preferred to consume healthy diets, including meat and eggs, containing fewer fats. Fatty acid (FA) compositions have become a major factor determining consumers’ decisions to accept or reject meat and eggs in their diets [1,2]. Hence, poultry farmers, particularly chicken and duck farmers, should be able to provide not only affordable and nutritious but also healthy meat and eggs containing more polyunsaturated fatty acids (PUFA) and lower cholesterol [3].

Improvement of the quality and nutrient composition of eggs through nutritional manipulation has been long known and studied [4,5]. Feeds with high PUFA content are one of the most common strategies applied to modify the fat profile of poultry products. Supplementation of canola oil, linseed oil, fish oil, flaxseed oil, and rapeseed oil in diets improved PUFA contents and lowered cholesterol levels in chicken eggs [6–8] and duck eggs [3].

The use of plant materials containing various types of specialized metabolites has also been revealed to have promising effects on improving the lipid profiles of poultry meat and eggs [9,10]. Secondary metabolites, including phenols, alkaloids, saponins, terpenes, lipids, and carbohydrates, are known to have antibiotic, antifungal, antiviral, antibacterial, anti-inflammatory, antitumor, antianaphylactic, antimutagenic, choleretic, and bronchodilatory properties [11–13].

Star gooseberry (*Sauropus androgynus*) is a tropical leafy vegetable that belongs to the family Phyllanthaceae and easily grows in areas with humid and high-temperature conditions throughout South, East, and Southeast Asia [14,15]. The leaves of this vegetable were found to be rich in vitamins A and C and other chemical compounds, including phytosterols, tannins, saponins, flavonoids, alkaloids, saponins, sterols, amino acids, proteins, carbohydrates, minerals, and kaempferol [12,16–18]. These compounds were known to have antioxidative, anticancerous, antidiabetic, antimicrobial, anti-inflammatory, antiobesity, lactation-inducing, antifungal, antialopecia, antianemia, antitussive, analgesic, and antiulcer properties [15,19].

Feeding dried star gooseberry leaf extract (DSGLE) diets to chickens and ducks resulted in meat and eggs containing lower cholesterol and higher PUFA. Meat from broiler chickens had lower cholesterol levels when the chickens were fed rations containing star gooseberry leaf meal, which was fermented [20,21] and extracted in warm (30ºC) and hot (60ºC) water [22]. The meat of ducks fed rations containing DSGLE was found to have higher PUFA, lower malondialdehyde (MDA), and lower cholesterol levels [2]. Egg yolk cholesterol levels were also found to be reduced with the inclusion of DSGLE in rations for laying hens [24], quails [25], and Mojosari ducks [26]. These findings have brought the notion to this study that including DSGLE in rations might improve the internal quality of duck eggs.

## Materials and Methods

### Ethical approval

The study was carried out at and supervised by the research committee of the Department of Animal Science, Faculty of Agriculture, Djuanda University, in compliance with Indonesian Government Regulation (PP) Number 95 Year 2012 on Veterinary Public Health and Animal Welfare.

### Animals, star gooseberry leaf extract, and rations 

A total of 100 9-month-old local ducks weighing 1,406.25 ± 211.32 gm were purchased from local farmers. The birds were randomly placed in 25 battery cages (4 birds each) and fed 5 treatment rations containing basal ration and the inclusion of DSGLE (0%, 0.5%, 1.0%, 1.5%, and 2.0%). A completely randomized design with five replicates was used. The formulation and nutrient composition of the treatment rations are shown in Table 1.

Star gooseberry leaf extract was obtained by referring to the procedures developed by Yasni et al. [27]. Fresh star gooseberry leaves (50 gm), which were soaked in ethanol (96%, 300 ml), were incubated in a water bath (60ºC), stirred slowly for 120 min, and filtered. The filtrate was let to evaporate until a volume of 100 ml was obtained. The evaporated filtrate was mixed with corn meal (90 gm), stirred slowly, and dried in an oven (50ºC–60ºC) for one and a half hours.

### Egg lipid profile 

Egg yolk and albumen were mixed and homogenized before they were used as samples in egg lipid profile determination. The egg fat content was measured based on the method developed by AOAC [28]. The egg sample was dried before its lipid content was extracted using an ether solution in a Soxhlet extractor for 16 h. The extracted lipid was later evaporated at 95ºC–100ºC and weighed. Fat content was calculated by subtracting the initial sample weight from the extract weight.

Egg cholesterol was analyzed using the method described by Kleiner and Dotti [29]. The homogenized egg sample (0.1 mg) and alcohol–ether solution (3:1; 12 ml) were centrifuged at 3,000 rpm for 15 min. The dried supernatant was dissolved in chloroform (5 ml), homogenized using a vortex, dissolved in anhydrous acetic acid (2 ml), and vortexed until the mixture became homogenous. Concentrated sulfuric acid (1 ml) was added to the mixture and stored in a dark room for 15 min. The absorbance measurement was conducted at a wavelength of 430 nm using a UV spectrophotometer.

**Table 1.** Feed composition and nutrient content of treatment rations.

	Treatment				
	R0	R1	R2	R3	R4
Feed/nutrient	Amount (%)				
Corn meal	61.00	61.00	61.00	61.00	61.00
Rice bran	8.00	8.00	8.00	8.00	8.00
Soybean cake meal	11.50	11.50	11.50	11.50	11.50
Fish meal	10.50	10.50	10.50	10.50	10.50
Premix	1.00	1.00	1.00	1.00	1.00
CaCO_3_	5.00	5.00	5.00	5.00	5.00
Crude palm oil	0.50	0.50	0.50	0.50	0.50
Dicalcium phosphate	2.50	2.50	2.50	2.50	2.50
DSGLE	0.00	0.50	1.00	1.50	2.00
Dry matter	89.12	89.08	89.04	89.00	88.97
Crude protein	16.31	16.35	16.40	16.44	16.49
Fibre	4.51	4.51	4.51	4.51	4.51
Crude fat	3.68	3.69	3.70	3.72	3.73
Ash	8.08	8.09	8.10	8.10	8.11

R0: 0% DSGLE, R1: 0.5% DSGLE, R2: 1.0% DSGLE, R3: 1.5% DSGLE, R4: 2.0% DSGLE.

The levels of MDA were measured using the method described in Esterbauer and Cheeseman [30] based on the ability of MDA to react with thiobarbituric acid (TBA). The egg sample was mixed with 2.5 ml of a cold (5ºC) phosphate buffer solution containing 11.5 gm/l potassium chloride (pH 7.4). The mixture was further centrifuged (4,000 rpm, 10 min), and the resultant clear supernatant (1 ml) was mixed with a mixture solution of cold 0.25 N (2.23 ml concentrated chloride acid/100 ml) chloride acid containing 15% (*w*/*v*) trichloroacetic acid, 0.38% TBA, and 0.5% butylated hydroxytoluene. The mixture was incubated at 80ºC for 1 h, cooled with running water, centrifuged (3,500 rpm for 10 min), and measured for its absorbance (532 nm).

The composition of FAs was determined based on the method used [31]. The extracted egg sample (100 gm) was homogenized in a chloroform and methanol solution (2:1). A NaCl 1.5% solution was added to the mixture to produce methyl esters. These methyl esters were later injected into a gas chromatography column by using a GC-2010 Plus-Shimadzu AOC 20i autoinjector, SP^®^-2560 capillary columns, L × I.D 100 m × 0.25 mm, df 0.20 μm, Supelco, Bellefonte, PA. The starting temperature was set at 70ºC. This temperature was gradually elevated within 2 min until 175ºC was reached. This elevation continued at 4ºC per minute until the final temperature of 215ºC was reached and maintained for 31 min.

FAs were identified through the comparison of the retention time of the sample methyl ester and the standard fatty acid methyl esters (FAMEs), including C4-C24 (FAME mix Sigma^®^), vaccenic acid C18: 1 trans-11 (V038-1G, Sigma^®^), C18: 2 trans-10 cis-12 (UC 61 M 100 mg), CLA C18: 2 cis-9, trans-11 (UC 60 M 100 mg) (Sigma^®^), and tricosanoic acid (Sigma^®^). FAs were measured by normalizing the area under the methyl ester curve using Shimadzu GC-2010 software 2.42. The amount of FAs was indicated by the percentage of total FAMEs.

### Egg yolk and albumen protein

The protein contents of egg yolk and albumen were determined using the AOAC method [28]. The dried egg yolk and albumen samples were each weighed (1.2 gm) and put in sample tubes. Sulfuric acid (20 ml) and two Kjeldahl tablets were added to each tube, and digestion was performed. After digestion, steam distillation was conducted to distill the ammonia from the sample into a boric acid solution before it was titrated with sulfuric acid.

## Results and Discussion

### Egg FAs

Eggs of ducks fed rations supplemented with DSGLE had significantly lower saturated fatty acid (SFA) (30.66%–32.53%) than those of control ducks (36.23%). The inclusion of DSGLE in rations by 1.5% and 2.0% increased egg PUFA from 2.29% (control) to 2.61% (R4) and 2.76% (R3). No significant differences were found in egg monounsaturated fatty acid (MUFA) contents. In this study, the FAs of the egg albumen and yolk were composed mainly of MUFA (40.19%–44.34%), followed by SFA (30.66%–36.22%), and PUFA (2.29%–2.76%) (Table 2). In the whole eggs of different varieties of Indian ducks, SFA, MUFA, and PUFA were found to be 45.49%–48.83%, 37.88%–45.00%, and 7.89%–10.57%, respectively [32]. Findings in Ismoyowati et al. [33] showed that the egg yolk of Tegal ducks in the control group had MUFA, SFA, and PUFA contents of 43.86%, 27.43%, and 11.22%, respectively.

**Table 2.** Egg FA compositions of ducks fed with DSGLE (%).

FAs	R0	R1	R2	R3	R4
Miristic	0.31 ± 0.02	0.28 ± 0.03	0.3 ± 0.03	0.31 ± 0.03	0.30 ± 0.04
Palmitic	24.66 ± 0.73^c^	20.62 ± 0.99^a^	20.87 ± 1.29^ab^	21.92 ± 1.35^ab^	22.61 ± 2.12^b^
Stearic	4.53 ± 0.34^cd^	3.23 ± 0.41^a^	4.73 ± 0.56^d^	3.97 ± 029^bc^	3.74 ± 0.50^ab^
Arachidic	6.73 ± 0.62	6.75 ± 0.58	6.32 ± 0.36	6.32 ± 0.38	6.28 ± 0.19
SFA	36.23 ± 1.43^b^	30.89 ± 1.68^a^	32.21 ± 1.53^a^	32.53 ± 1.47^a^	30.66 ± 0.67^a^
Palmitoleic	2.39 ± 0.31	3.26 ± 0.49	2.65 ± 0.26	32.53 ± 1.47	3.18 ± 0.38
Oleic	39.79 ± 1.99^ab^	39.37 ± 1.34^ab^	41.69 ± 0.52^b^	37.86 ± 2.02^a^	37.89 ± 1.99^a^
MUFA	42.18 ± 2.23^ab^	42.63 ± 1.04^bc^	44.34 ± 0.49^c^	41.11 ± 1.91^ab^	40.19 ± 1.13^a^
Linoleic	0.03 ± 0.01^a^	0.05 ± 0.01^cd^	0.05 ± 0.01^bc^	0.04 ± 0.11^ab^	0.06 ± 0.01^d^
Linolenic	0.12 ± 0.09^ab^	0.07 ± 0.03^a^	0.20 ± 0.11^b^	0.11 ± 0.01^ab^	0.12 ± 0.04^ab^
Arachidonic	2.14 ± 0.05^a^	2.22 ± 0.08^a^	2.10 ± 0.19^a^	2.56 ± 0.28^b^	2.48 ± 0.12^b^
PUFA	2.29 ± 0.14^a^	2.34 ± 0.08^a^	2.35 ± 0.28^a^	2.76 ± 0.17^b^	2.61 ± 0.11^b^

Different superscripts in the same rows indicate significant differences (*p *< 0.05). R0: 0% DSGLE, R1: 0.5% DSGLE, R2: 1.0% DSGLE, R3: 1.5% DSGLE, R4: 2.0% DSGLE, SFA: saturated fatty acids, MUFA: monounsaturated fatty acids, PUFA: polyunsaturated fatty acids.

In this study, the inclusion of DSGLE in the ration reduced SFA contents from 36.25% to 30.66% and increased PUFA contents from 2.29% to 2.61% in ducks’ eggs. Ducks fed rations containing DSGLE-produced eggs with lower palmitic and stearic acid contents (Table 2). In another study, the use of DSGLE in broiler ducks resulted in meat containing lower SFA (19.14%) and higher PUFA (40.50%) contents than those of control ducks (21.48% and 37.27%, respectively) [23]. The effects of DSGLE inclusion on the profile of FAs in duck eggs in this study might be attributed to the metabolites contained in DSGLE. The leaves of star gooseberry plants were known to contain FAs, which mainly included oleic, linoleic, and linolenic acids [19,34]. It has been known that dietary fat and FA profiles play a more significant role in lipid metabolism and FA synthesis in animals than genetic factors. The FA profiles of meat and eggs are affected by the FA contents of the animals’ diet [35]. In hen eggs, lipids are mostly found in the yolk, which is composed of 60% lipids, while less than 0.1% are contained in the whites. These egg lipids are mostly in the forms of triacylglycerol (65%–68%), phospholipids (29%–32%), and cholesterol (<5%) [36,37]. In chicken and duck, lipids are mainly synthesized from FAs released from diets and adipose tissue in the liver through a de novo synthesis pathway [36]. Using flaxseed high in α-linolenic acid (ALA) content by 15% in rations enhanced total PUFA contents from 14.40% to 18.39% in the egg yolk of geese [38]. Linear effects of plant materials containing high ALA on egg FA contents of chickens fed purslane leaves [39], thyme and fennel extracts [8], linseed and soybean oil [40], and ducks fed leaves of *Eucommia ulmoides *oliv [10] were also observed.

In addition, star gooseberry leaves were found to have many other metabolites, including polyphenols (quercetin and kaempferol), carotenoids, ascorbic acid, anthocyanin, and squalene [15,16,19]. Along with FA and their derivatives, these metabolites had antioxidant properties by modifying the intracellular production of reactive oxygen species [34]. This antioxidant activity might help prevent PUFAs from oxidizing, as indicated by lower MDA levels found in eggs of ducks fed DSGLE (). The antioxidative effects of star gooseberry leaf meal were also observed in duck and broiler meat [21–23] and chicken and quail eggs [24,25].

### Egg MDA, thiobarbituric acid reactive substances (TBARS), and cholesterol

MDA, an indicator of peroxidation of mono-and PUFA of lipid substrates by free radicals in the body, can be measured through the TBARS assay. Peroxidation of FAs is often associated with oxidative stress in body organs [41,42]. In this study, the inclusion of DSGLE in the ration was found to significantly lower MDA contents from 6.07 to 4.92 µg/gm (19%) in egg yolk and from 1.50 to 0.62 µg/gm (59%) in egg white. This was confirmed by significantly increased TBARS values of the egg, from 0.93 to 1.65 (77%) ().

The antioxidative effects of dietary plant materials on the antioxidant status of animal products have been found in other studies. Dietary supplementation of resveratrol, a natural polyphenol commonly found in grapes, by 200–400 mg of peanut and turmeric lowered quail egg MDA levels from 0.21 to 0.15 μg/gm (29%) [41]. A reduction of MDA levels from 0.36 to 0.11 gm/100 gm was also observed in the meat of ducks fed rations containing star gooseberry leaf meal [23]. Although no quantification of phenolic and other secondary metabolites was conducted in this study, results of a recent study by Hikmawanti et al. [43] showed that ethanolic extract (50% ethanol solvent) of star gooseberry leaf had a total phenolic content of 42.18 mgGAE/gm and a total flavonoid content (TFC) of 11.18 mgQE/gm, [16] performed ethanol extraction of star gooseberry leaves and observed that the leaves contained 142.64 mg/100 gm fresh weight of TFC consisting mainly of quercetin and kaempferol. Regarding the antioxidant activity, these secondary metabolites of star gooseberry leaves were shown to have reduction activity against (2,2-Diphenyl-1-picrylhydrazy) radicals (IC50) of 49.62–88.33 ppm [19,43]. In addition, the leaves of star gooseberry contain high levels of vitamins C and E, which are well known for their excellent antioxidative properties [19,25,44].

**Table 3.** Egg MDA, TBARS, and cholesterol contents of ducks fed with DSGLE.

	R0	R1	R2	R3	R4
Yolk MDA (µg/gm)	6.07 ± 1.27^a^	5.31 ± 0.62^b^	4.93 ± 0.65^b^	4.95 ± 0.56^b^	4.92 ± 0.30^b^
White MDA (µg/gm)	1.50 ± 0.70^b^	1.47 ± 0.68^b^	1.31 ± 0.64^b^	0.95 ± 0.57^ab^	0.62 ± 0.17^a^
TBARS	0.93 ± 0.33^a^	1.22 ± 0.46^ab^	1.58 ± 0.63^bc^	1.61 ± 0.34^bc^	1.65 ± 0.18^c^
Cholesterol (mg/gm)	30.67 ± 9.74^b^	30.55 ± 4.38^b^	25.83 ± 4.03^a^	23.07 ± 6.50^a^	21.94 ± 2.51^a^

Different superscripts in the same rows indicate significant differences (*p *< 0.05). R0: 0% DSGLE, R1: 0.5% DSGLE, R2: 1.0% DSGLE, R3: 1.5% DSGLE, R4: 2.0%.

**Table 4.** Protein content (%) of egg white and yolk of ducks fed with DSGLE.

	R0	R1	R2	R3	R4
Egg white	13.58 ± 3.14^b^	10.11 ± 2.52^a^	13.01 ± 0.33^b^	12.45 ± 0.42^b^	12.31 ± 0.52^b^
Egg yolk	17.47 ± 0.61^c^	16.12 ± 0.54^b^	16.46 ± 0.41^b^	15.43 ± 1.06^a^	16.35 ± 0.69^b^

Different superscripts in the same rows indicate significant differences (*p *< 0.05). R0: 0% DSGLE, R1: 0.5% DSGLE, R2: 1.0% DSGLE, R3: 1.5% DSGLE, R4: 2.0%.

The egg cholesterol levels of ducks fed rations supplemented with DSGLE were significantly lower than those of the control group (Table 3). Eggs of ducks fed a ration containing 2% DSGLE had a cholesterol content of 21.94 mg/gm, about 28.5% lower than those of control ducks (30.67 mg/gm). The inclusion of star gooseberry leaf meal and extract has been found to reduce the cholesterol levels in chicken whole egg and yolk egg [24,45], duck egg [26], duck meat [23], and broiler chicken meat [20].

The cholesterol-reducing effects of DSGLE in animal products might be attributable to the phytosterols contained in DSGLE. Sterols are a class of important lipids found in animals, plants, and fungi in different forms, including cholesterol, phytosterols, and ergosterol, respectively [46]. More than 250 kinds of phytosterols have been recognized, yet three of them, including campesterol, stigmasterol, and sitosterol, are found the most (>95%) in plant materials [47–49]. Star gooseberry leaves contained very high levels of phytosterols (2.43/100 gm dry or 466 mg/100 gm fresh), composing stigmasterol and sitosterol [25,50]. The effects of phytosterols on reducing cholesterol in animal blood serum and products were also observed in animals used in laboratory and clinical trials [51], confirming that phytosterols in plant materials could be appropriately used to produce healthy animal products containing low cholesterol.

The hypocholesterolemic effects of phytosterols have been recognized for a long time [51]. Some mechanisms of phytosterol interference in cholesterol synthesis were proposed. In earlier times, phytosterols were thought to compete with cholesterol to be incorporated into mixed micelles and chylomicrons absorbed into the intestinal wall. However, it was then proven that the simultaneous existence of phytosterols and cholesterol did not hamper cholesterol absorption in the intestinal lumen [52]. Later, a reduction in the efficiency of cholesterol absorption was observed as a result of the regulation of the activation of liver X receptor-alpha (LXR-α) Pick C1-Like-1 and ATP-binding cassette subfamily G members (G8) through the activation of LXR-α. The interference of cholesterol esterification and incorporation into chylomicrons via Acyl CoA cholesterol acyltransferase isoform, microsomal triglyceride transfer protein, and apolipoprotein B48 was proposed as another mechanism of interference of dietary phytosterol on cholesterol synthesis [49].

### Egg proteins

Eggs are excellent sources of diverse nutrients, including proteins, lipids, minerals, and vitamins, which support the development of embryos and fulfill humans’ requirements for nutrients. These nutrients are available at a well-balanced and affordable price [53,54].

Eggs of ducks fed a ration containing DSGLE, in this study, had lower egg white and egg yolk protein contents (12.31% and 16.35%) than those of ducks in the control group (13.58% and 17.47%) (Table 4). Although the protein content of egg white and egg yolk was lowered in ducks fed DSGLE, the figures were still higher than those of egg white (8.60%–10.50%) and egg yolk (14.10%–16.0%) of various kinds of ducks [55,56]. In another study in chickens, DSGLE feeding did not change egg protein contents [24].

Secondary metabolites, including polyphenols contained in star gooseberry leaves [15], might be attributable to the findings that eggs of ducks fed DSGLE in this study had egg white and egg yolk with lower protein contents. It has been known that polyphenols bind with proteins and proteolytic enzymes, and this binding may change the structure and bioavailability of proteins [57]. Bindings of phenols and endogenous proteins, including gastric and intestinal mucus, digestive enzymes, and protein saliva, affect protein digestibility and metabolism. Lowered proteolytic activities are also observed as an effect of phenolic compounds [58]. However, no dietary protein digestibility assessment was conducted in this study.

## Conclusion

The inclusion of DSGLE in rations reduced the SFA contents and increased the PUFA contents, thus lowering the cholesterol level and protein contents of duck eggs. It also protected the eggs from oxidative damage. The results of this study reinforced the advantageous effects of DSGLE on the creation of healthy poultry products, particularly eggs.

## List of Abbreviations

ALA, α-linolenic acid; DSGLE, Dried star gooseberry leaf extract; FA, Fatty acid; FAME, Fatty acid methyl ester; MDA, Malondialdehyde; MUFA, Monounsaturated fatty acid; PUFA, Polyunsaturated fatty acid; SFA, Saturated fatty acid, TBA, Thiobarbituric acid; TBARS, Thiobarbituric acid reactive substances; TFC, Total flavonoid content.
